# Phase transitions in two tunnel-coupled HgTe quantum wells: Bilayer graphene analogy and beyond

**DOI:** 10.1038/srep30755

**Published:** 2016-08-01

**Authors:** S. S. Krishtopenko, W. Knap, F. Teppe

**Affiliations:** 1 Institute for Physics of Microstructures RAS, GSP-105, 603950, Nizhni Novgorod, Russia; 2 Laboratoire Charles Coulomb (L2C), UMR CNRS 5221, Universite Montpellier, 34095 Montpellier, France; 3 Institute of High Pressure Physics, Polish Academy of Sciences, Sokolowska 29/37 01-142 Warsaw, Poland

## Abstract

HgTe quantum wells possess remarkable physical properties as for instance the quantum spin Hall state and the “single-valley” analog of graphene, depending on their layer thicknesses and barrier composition. However, double HgTe quantum wells yet contain more fascinating and still unrevealed features. Here we report on the study of the quantum phase transitions in tunnel-coupled HgTe layers separated by CdTe barrier. We demonstrate that this system has a 3/2 pseudo spin degree of freedom, which features a number of particular properties associated with the spin-dependent coupling between HgTe layers. We discover a specific metal phase arising in a wide range of HgTe and CdTe layer thicknesses, in which a gapless bulk and a pair of helical edge states coexist. This phase holds some properties of bilayer graphene such as an unconventional quantum Hall effect and an electrically-tunable band gap. In this “bilayer graphene” phase, electric field opens the band gap and drives the system into the quantum spin Hall state. Furthermore, we discover a new type of quantum phase transition arising from a mutual inversion between second electron- and hole-like subbands. This work paves the way towards novel materials based on multi-layered topological insulators.

Low energy band structure in graphene is formed by two spin-degenerate massless Dirac cones at two inequivalent valleys, giving rise to four massless Dirac cones in total^1^,^2^. With one additional graphene layer added, bilayer graphene (BG) has an entirely different band structure. Most notably, symmetric BG is a zero-band gap semiconductor with quadratic energy-momentum dispersion^3^. However, its band gap is continuously tunable through an electrical field applied perpendicularly to the sample^4^,^5^. The electronic states in BG are also four-fold degenerate, taking into account both spin and valley degeneracies. Recently, it has been shown that HgTe quantum wells (QWs) with vanishing band gap possess a single spin-degenerate Dirac cone at the Brillouin zone center^6^,^7^,^8^,^9^ and thus can be considered as the “single-valley” analog of graphene in two-dimensional (2D) semiconductor heterostructures.

The central feature of the HgTe QWs is the possibility of band inversion. The barrier material (e.g., CdTe) has a normal band ordering, with the *s*-type Γ_6_ band lying above the *p*-type Γ_8_ band, while in the well material (HgTe) the Γ_6_ band lies below the Γ_8_ band, corresponding to an inverted band ordering. If the thickness *d* of the HgTe/CdTe QWs does not exceed a critical value *d*_*c*_, at the Γ point, the first conduction subband (E1) is electron-like, i.e. it is formed by a linear combination of the |Γ_6_, *m*_*J*_ = ±1/2〉 and |Γ_8_, *m*_*J*_ = ±1/2〉 states, while the first valence subband (H1) arises from the |Γ_8_, *m*_*J*_ = ±3/2〉 states, corresponding to the heavy-hole band. In wide HgTe QWs (*d* > *d*_*c*_), the *E*1 subband falls below the *H*1 subband. The inversion between *E*1 and *H*1 subbands leads to the formation of a 2D time-reversal invariant topological insulator (TI)^10^ denoted the quantum spin Hall (QSH) state^6^,^7^ with dissipation less edge channel transport at zero external magnetic field^11^. At critical QW thickness *d*_*c*_, corresponding to topological phase transition between a TI and a trivial band insulator (BI), a low-energy band structure mimics a massless Dirac cone at the Γ point^6^.

Two tunnel-coupled HgTe QWs of critical thickness may, therefore, share some properties of natural BG. If each of the HgTe QW has *d* > *d*_*c*_, this system offers a practical realization of tunnel-coupled layers of 2D TI. Moreover, the strained HgTe thick films have been proven to be three-dimensional (3D) TIs^12^,^13^. Therefore, a wide double HgTe QW can be considered as two thick layers of 3D TI separated by BI (CdTe material) with the surface states arising at the interfaces^14^. A tensile strain in the HgTe films, which opens a topological gap, is induced by the difference in the lattice parameters of HgTe and CdTe. Thus, double HgTe QWs is a realistic system, which potentially paves the way towards physics of multi-layered topological insulator materials.

So far, there are just a few works devoted to double HgTe QWs^15^,^16^,^17^. All these works are based on the approach, in which each QW is described within Bernevig-Hughes-Zhang (BHZ) model^6^ coupled by a spin-conserved tunneling Hamiltonian. The latter will be shown to be irrelevant on double HgTe QWs, which feature a much richer physics than previously assumed.

In our theoretical investigation of double HgTe QWs (see Fig. 1), we first start from band structure calculations on the basis of an eight-band Kane model^18^. By using realistic material parameters, we obtain the phase diagram for symmetrical double QW as a function of the layer thicknesses. We discover a specific metal phase, in which the band properties in perpendicular magnetic and electric fields are very similar to the ones of natural BG. Then, we deduce an effective 2D Hamiltonian, involving two electron-like (*E*1, *E*2) and two hole-like (*H*1, *H*2) subbands, to describe quantum phase transitions in the system. On the basis of such a simplified Hamiltonian, we calculate dispersion of edge states in different phases. We show that inversion between *E*2 and *H*2 subbands induces an additional pair of helical edge states, putting the system into BI phase even in the case of inverted band structure. The crossing between *E*2 and *H*2 levels yields a Dirac cone in the Γ point of the Brillouin zone.

Considering double HgTe QW, shown in Fig. 1, as a whole system and by using expansion in the plane-wave basis set (see Supplementary Information), we calculate energy dispersions of electronic subbands. Figure 2A,B show positions of electron-like and hole-like subbands at ***k*** = 0 as a function of middle barrier thickness *t*. Intuitively, it is clear that at infinitely large barrier, calculated states correspond to the subband positions in two separated HgTe QWs, while at finite values of *t* each pair of the subbands is connected with even-odd state splitting in double QWs. If *t* tends to zero, the energy values transform into positions of electronic subbands in single HgTe QWs of 2*d* thickness. It is seen that the splitting between electron-like levels exceeds significantly the one of the hole-like levels. This is due to significant difference in effective masses at ***k*** = 0 in electron-like and hole-like subbands, which values determine the tunnel-coupling between the states and their even-odd splitting at given *t*.

There are two types of the band structure ordering, which arises depending on the HgTe layers thickness *d*. The first case shown in Fig. 2A takes place if *d* varies in the range from *d*_*c*_/2 to *d*_*c*_, where *d*_*c*_ corresponds to the thickness of single QW with Dirac cone at the Γ point. In this case *E*2 subband always lies above *H*2 subband and inversion between *E*1 and *H*1 subband can take place. At higher values of *t*, the *E*1 subband gains a higher energy than the *H*1 subband, and the system has the normal band structure. When the thickness *t* is decreased, the energy of the *E*1 subband reduces, whereas the *H*1 subband energy practically does not change. The different dependence of *E*1 and *H*1 subbands on *t* implies that at some barrier thicknesses the band gap closes. In fact, the crossing point between *E*1 and *H*1 subbands yields a low-energy band structure with Dirac cone in the vicinity of ***k*** = 0 (Supplementary Information). If the values of *t* are small enough to induce a gap between *H*1 and *H*2 subbands, it implies a quantum phase transition between BI and TI states, as in single HgTe QW^6^. However, if *H*1 and *H*2 subbands coincide at ***k*** = 0, it gives rise to an additional “massive” branch of valence subband at the crossing point, similar to the pseudospin-1 Dirac-Weyl 2D systems^19^, as well as a quantum phase transition into a specific metal phase with the following subband ordering: *E*2-*H*1-*H*2-*E*1. The reasons to assign this metal phase as a BG phase are discussed later.

The second type of band structure ordering, shown in Fig. 2B, is realized when *d* > *d*_*c*_. In this case *E*1 subband always lies below *H*1 subband, i.e. they are inverted. At lower barrier thickness, *E*2 subband lies above *H*2 subband, while at high values of *t*, they swap their positions. At specific values of *t*, *E*2 and *H*2 subbands cross, which also leads to the appearance of a Dirac cone at the Γ point. As for the case shown in Fig. 2A, additional “massive” branch arises as well. However, such branch corresponds to conduction *H*1 subband. Up to date, it was never realized that crossing between *E*2 and *H*2 subbands also induces a quantum phase transition. In this work we explicitly demonstrate it for the first time. In particular, we show that this crossing point in double HgTe QWs correspond to the quantum phase transition between BG and BI phase.

Figure 2C shows the phase diagram, in which two bold lines correspond to the Dirac cones at the Γ point. The left one results from the crossing between *E*1 and *H*1 subbands, while the right-side curve is connected with the crossing of *E*2 and *H*2 levels. If the middle barrier is thin enough, a gap between *H*1 and *H*2 subbands opens, and the inversion between *E*1 and H1 levels induces a quantum phase transition between BI and TI phases. The latter is shown by orange region. For relatively wide QWs, the so-called semimetal (SM) phase, corresponding to the white-striped region, is implemented. It is characterized by a vanishing indirect band gap, when the side maximum of the valence subband exceeds in energy the conduction subband bottom. This phase arises in single HgTe QWs as well when the thickness goes beyond some critical value, denoted in Fig. 2C by *d*_*SM*_^20^,^21^. BG metal phase mentioned above corresponds to the blue region in Fig. 2C. Dispersion curves at various values of *d* and *t* in the vicinity of phase transitions between BI, BG and SM phases are provided in Supplementary Information.

Let us now explain the reasons to call this specific metal phase with the ordering of electronic subbands *E*2-*H*1-*H*2-*E*1 a BG phase. First, we consider in details the case when both QWs have a critical thickness *d*_*c*_ ≈ 6.5 nm, which at infinitely large barrier corresponds to two Dirac cones. The presence of the transparent barrier, for example of *t* = 3 nm, turns from two Dirac cones in the vicinity of the Γ point into a band structure very similar to the one of natural BG^3^ (c.f. Fig. 3A). In particular, it consists of two gapless isotropic parabolas, formed by *H*1 and *H*2 subbands. Moreover, as in BG in which non-zero band gap can be induced by breaking the inversion symmetry of two monolayers, in double HgTe QWs it could be obtained by using QWs of different thickness or by adding of one-side chemically doping. Moreover, the potential of a continuously tunable band gap through an electric field applied perpendicularly to the sample plane is of particular importance.

The double HgTe QW in BG phase also holds this property. Figure 3B displays energy dispersion for BG phase in perpendicular electric field of 20 kV/cm. Even in this case the dispersion curves are very similar to the band structure of natural BG in external electric field^3^,^4^. However, strong spin-orbit interaction in HgTe layers removes the spin degeneracy away from the Γ point due to Rashba effect^22^. Figure 3C shows the band gap values in the double QWs in BG phase as a function of applied electric field. Indeed, the band gap in BG phase is electrically-tunable, as it is in natural BG. However, its dependence on electric field has non-monotonic behaviour in double HgTe QWs. The reason for this is related with the additional side maximum of valence subband, which is also responsible for the formation of SM phase in zero electric field (see the diagram, shown in Fig. 2C). The top of the side maximum is increasing with the strength of electric field, and the band gap reduces (see the inset, shown in Fig. 3C). As a result, the band gap is closed in high enough electric field, giving rise to the formation of the SM phase. A critical electric field, corresponding to arising of indirect-band gap, coincides with the maximum value of Δ, shown in Fig. 3C.

Another characteristic of natural BG is the unconventional quantum Hall effect, related with the absence of zero-Landau level (LL) plateaus in Hall conductivity^23^. For natural BG, plateaus in the Hall conductivity *σ*_*xy*_, occur at integer multiples of 4*e*^*2*^*/h*. This is similar to a conventional semiconductor with level degeneracy *g* = 4 arising from the spin and valley degrees of freedom. Deviation from the conventional case occurs at low density, where there is a step in *σ*_*xy*_ of height 8*e*^*2*^*/h* across zero density, arising from the eightfold degeneracy of the zero-energy LL. This specific LL is formed by atomic orbitals of different sublattice sites from both layers^24^.

Figure 3D shows LL fan chart in double HgTe QW with BG phase, calculated within the eight-band Kane model. In the BG phase, in perpendicular magnetic field a specific zero-mode LL with degeneracy two times higher than other LLs also arises. This zero-mode LL is shown in Fig. 3D, by bold orange line. For double HgTe QW, plateaus in the Hall conductivity are expected to occur at integer numbers of *e*^*2*^*/h*. However, the doubled degeneracy of zero-mode LL requires twice as many carriers to fill them, so the transition between the corresponding plateaus should be twice as wide in density, and the step in *σ*_*xy*_ between the plateaus are expected to be twice as high, 2*e*^*2*^*/h* instead of *e*^*2*^*/h*. Since the zero-mode LL in double HgTe QW is formed by the states of both *H*1 and *H*2 subbands, the presence of inversion asymmetry of two HgTe layers not only opens the band gap between *H*1 and *H*2 subbands (see Fig. 3B) but also splits the zero-mode LLs, removing the double degeneracy order. Therefore, we also expect recovering of sequence of equidistant plateaus in the Hall conductivity as it is for gate-biased natural BG^5^.

Besides the zero-mode LL, there are two additional specific LLs, which in high magnetic fields are formed only by the states from *E*1 and *E*2 subbands. These LLs are shown in Fig. 3D by blue curves. In moderate magnetic fields, these *E*1 and *E*2 LLs are mixed with the states from *H*2 and *H*1 subbands respectively. It results in anticrossing between *E*1 (*E*2) LL and LLs from *H*2 (*H*1) subband, which is clearly seen in magnetic fields below 1 T. The crossing between the *E*1 and zero-mode LL, arising at critical magnetic field *B*_*c*_ ~ 3.5 T, corresponds to the transition from inverted into normal band structure, similar to this observed in single HgTe QW^7^.

We have considered in details the case of double HgTe QW, when both HgTe layers have a critical thickness *d*_*c*_. However, all the mentioned properties of BG phase hold for any double QW with the values of *d* and *t*, corresponding to the blue region in Fig. 2C. The only difference is the ratio *M*_*1*_ over *M*_*2*_, where *M*_*1*_ parameter describes the energy gap between *E*1 and *H*1 subbands, while *M*_*2*_ corresponds to the half of the gap between *E*2 and *H*2 levels. If *d* < *d*_*c*_, 2*M*_*2*_ exceeds 2*M*_*1*_, while in the opposite case of *d* > *d*_*c*_, the gap between *E*2 and *H*2 subbands is lower than 2*M*_*1*_. An amazing property of double HgTe QW is that it shares some characteristics of natural BG even in the BI phase at *d* > *d*_*c*_. In particular, the possibility of tuning the band gap by electric field and of observing the unconventional double step in plateaus in the Hall conductivity still persists.

We now discuss quantum phase transitions in double HgTe QWs. For this purpose, we derive an effective 2D Hamiltonian, which describes the band structure in the vicinity of ***k*** = 0 in the phases shown in Fig. 2C. To infer this simplified model, we start from eight Bloch basic states, combined into an eight-component spinor:


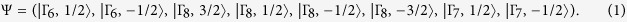


For the QWs grown in [001] direction, projection *m*_*J*_ of total angular momentum at ***k*** = 0 on the growth direction is still a good quantum number. At the Γ point all QW subband states are formed by linear combination of the mentioned eight bulk bands. To describe BI, TI and BG phases, one should consider *E*1, *E*2, *H*1 and *H*2 subbands (see Fig. 2A,B). At ***k*** = 0, the |*E*1, ±〉 and |*E*2, ±〉 subband states are formed from the linear combination of the |Γ_6_, *m*_*J*_ = ±1/2〉, |Γ_7_, *m*_*J*_ = ±1/2〉 and |Γ_8_, *m*_*J*_ = ±1/2〉, while the |*H*1, ±〉 and |*H*2, ±〉 QW states are formed from the |Γ_8_, *m*_*J*_ = ±3/2〉 states. Away from the Γ point, the *E*1, *E*2, *H*1 and *H*2 states are mixed. To construct the effective Hamiltonian one should carefully take into account different parities of the envelope function components from |Γ_6_, ±1/2〉, |Γ_7_, ±1/2〉, |Γ_8_, ±1/2〉 and |Γ_8_, ±3/2〉 bulk bands into formation of given subband state^25^. For instance, *E*1 state is formed by even function corresponding to |Γ_6_, ±1/2〉 band and by odd envelope functions corresponding to |Γ_7_, ±1/2〉 and |Γ_8_, ±1/2〉 bands, while parities of their contributions into *E*2 state are changed. In previous works^15^,^16^,^17^, this point was missed; it results in the wrong description of double HgTe QWs. Detailed explanation is given in Supplementary Information.

After straightforward calculations, we obtain the following form of the effective 2D Hamiltonian for the *E*1, *E*2, *H*1, *H*2 states, expressed in the basis of two Kramer’s sets |*E*1, +〉, |*H*1, +〉, |*H*2, −〉, |*E*2, −〉 and |*E*2, +〉, |*H*2, +〉, |*H*1, −〉, |*E*1, −〉:


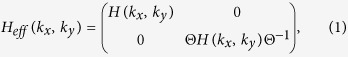


where Θ is a “time reversal” operator, given by


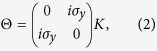


with *K* stands for complex conjugation and *σ*_*y*_ is one of the Pauli spin matrices. Each block in (1) is described by a four-component spinor with pseudospin *J* = 3/2 degree of freedom (see Supplementary Information). *H*(*k*_*x*_, *k*_*y*_) is written as


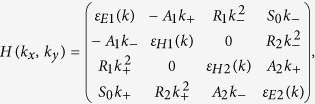



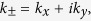



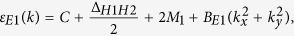



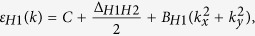



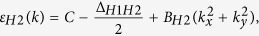



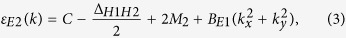


Here, *k*_*x*_ and *k*_*y*_ are momentum components in the plane of double QW, and *C*, *M*_*1*_, *M*_*2*_, *A*_*1*_, *A*_*2*_, *B*_*E1*_, *B*_*H1*_, *B*_*H2*_, *B*_*E2*_, *Δ*_*H1H2*_, *R*_*1*_, *R*_*2*_, *S*_*0*_ are specific heterostructure constants, being defined by QW geometry and materials. The Hamiltonian *H*_*eff*_ (*k*_*x*_*, k*_*y*_) has block-diagonal form because we keep the inversion symmetry and axial symmetry around the growth direction (see Supplementary Information). We note that *H*_*eff*_ (*k*_*x*_*, k*_*y*_) is valid for any values of *d* and *t*. In particular for *t* = 0, it describes energy dispersion in the vicinity of the Γ point in single HgTe QW beyond the BHZ model. Parameters *Δ*_*H1H2*_, *R*_*1*_, *R*_*2*_, *S*_*0*_ significantly depend on *t*, and all tend to zero at large middle barrier thickness, while *B*_*E1*_ = *B*_*E2*_ coincides. As it is easy to see, in this case the system is described by two non-interacting BHZ models, written for two pairs formed by *E*1, *H*1 and *E*2, *H*2 subbands. We note that *B*_*H1*_ = *B*_*H2*_ if *Δ*_*H1H2*_ = *0*.

The most important quantities in *H*(*k*_*x*_, *k*_*y*_) are two mass parameters *M*_*1*_ and *M*_*2*_. Almost all phases in Fig. 2C can be grouped into three types according to the sign of *M*_*1*_ and *M*_*2*_. The BI phase at normal band structure, shown in the left part of the diagram, corresponds to positive values of *M*_*1*_ and *M*_*2*_. The BG and TI phases arise if *M*_*1*_ < 0 and *M*_*2*_ > 0, and the difference between these phases is connected with *Δ*_*H1H2*_, which equals to zero in the case of BG phase. The second BI phase, shown in the right from the BG phase, corresponds to negative values of *M*_*1*_ and *M*_*2*_, and *Δ*_*H1H2*_ = 0. The SM phase, arising at all values of *M*_*2*_ and *M*_*1*_ > 0, cannot be described within the simplified model because it is only valid near the Γ point, while the SM phase is formed by non-local overlapping of the valence band and conduction band bottom^20^,^21^. Comparison between calculations of subband curves, performed within the simplified model and on the basic of an eight-band Kane Hamiltonian for different phases is given in Supplementary Information.

Zero values of *M*_*1*_ and *M*_*2*_ both conform to quantum phase transitions at which Dirac cone at the Γ point occurs. The left and right bold black curves in the phase diagram, shown in Fig. 2C, are related with *M*_*1*_ = 0 and *M*_*2*_ = 0 respectively. Figure 4 presents energy dispersion for the bulk and edge states in various phases in double HgTe QWs obtained within the simplified model. Dispersion of the bulk states are shown in black, while the orange and blue curves are the edge states, described by different blocks of *H*_*eff *_(*k*_*x*_*, k*_*y*_). The electrons in the edge states, marked by different colors, move in opposite directions. Figure 4 demonstrates that each change of sign of mass parameter *M*_*1*_ or *M*_*2*_ yields a pair of helical edge states, providing a quantum phase transition at *M*_*1*_ = 0 or *M*_*2*_ = 0. In particular, BG phase arising at *M*_*1*_ < 0 and *M*_*2*_ > 0 has a single pair of the edge states, coexisting with the gapless bulk states. If *M*_*1*_ < 0 and *M*_*2*_ = 0, the double HgTe QW mimics a 2D system with the presence of both bulk and edge massless fermions, which energy dispersions are shown in Fig. 4C.

Figure 4D shows special case with inversion between *E*2 and *H*2 subbands, which was not considered in 2D systems so far. It perfectly illustrates that each crossing between electron-like and hole-like levels of higher indexes, also results in the appearance of a pair of helical edge states. Topological properties of corresponding insulator phase are determined by amount of inverted levels, which are connected with the number of pairs of the edge states. In the particular case of inversion of both *E*1 with *H*1 subband and *E*2 with *H*2 subband, the terms proportional to *R*_*1*_, *R*_*2*_ and *S*_*0*_ induce the coupling between counter-propagating states with the same spin orientation. The latter could be interpreted as a spin-dependent tunneling between two layers of 2D TI (see Supplementary Information). It is clear from the inset that such tunnel-coupling opens the gap in the energy spectrum of the edge states even without external one-particle scattering processes. Thus, we prove that double HgTe QW with *M*_*1*_ < 0 and *M*_*2*_ < 0 has a BI phase.

Let us now remark the main difference between BG phase in double HgTe QW and natural BG. The electrons in natural BG are chiral particles with *S* = 1/2 pseudospin of freedom^23^,^24^ and pseudospin winding number of 2^25^. It results from the commutation of operator 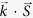 with the low-energy Hamiltonian of natural BG. As it is shown above, the simplest Hamiltonian, required for the description of bulk and edge states in the BG phase, has *J* = 3/2 pseudospin of freedom. Moreover, it can be shown that operator 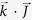 does not commute with *H*(*k*_*x*_, *k*_*y*_), proving the non-chiral character of electron in double HgTe QWs. Thus, even though BG phase mimics some characteristics of natural BG, it is a novel and fascinating state of matter, exhibiting the coexistence of gapless bulk states and spin-polarized edge channels.

Since we have demonstrated the existence of gapless spin-polarized counter-propagating edge channels in double HgTe QWs, they should exhibit QSH effect in both TI (*Δ*_*H1H2*_ ≠ 0) and BG (*Δ*_*H1H2*_ = 0) phases. Intuitively it is clear that QSH effect in the TI phase could be observed on the sample with a six-terminal Hall bar, as it has been previously proposed for single QW^6^,^7^. However, the measurements of two-terminal conductance in the BG phase contain contributions both from the bulk states and from the helical edge modes. To separate these contributions, we propose to introduce inversion asymmetry between the HgTe layers. If the band gap for the bulk states is open, for example by an external electric field, the system is driven to the TI regime, and the detection of the QSH effect becomes possible^6^.

## Additional Information

**How to cite this article**: Krishtopenko, S. S. *et al*. Phase transitions in two tunnel-coupled HgTe quantum wells: Bilayer graphene analogy and beyond. *Sci. Rep*. **6**, 30755; doi: 10.1038/srep30755 (2016).

## Supplementary Material

Supplementary Information

## Figures and Tables

**Figure 1:**
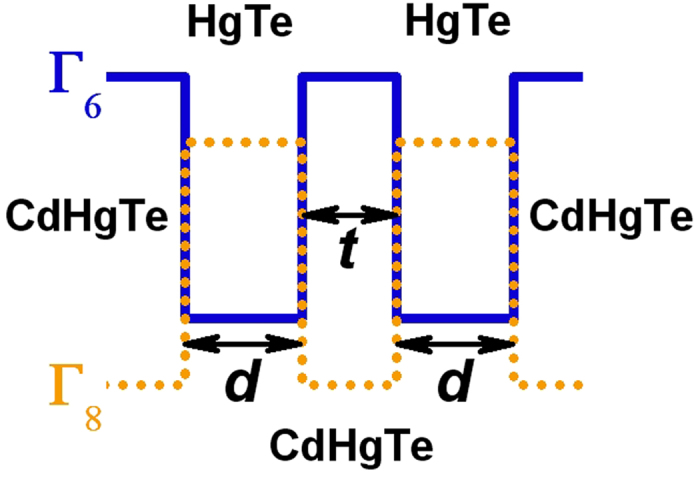
Schematic representation of the double HgTe/CdHgTe QW. Here *d* is the thickness of HgTe layers and *t* is the middle barrier thickness. Further, we consider the double QW grown on CdTe buffer in (001) crystallographic direction. The concentration of mercury in the top, middle and lower barriers is assumed to be equal to 0.3^7^,^8^.

**Figure 2:**
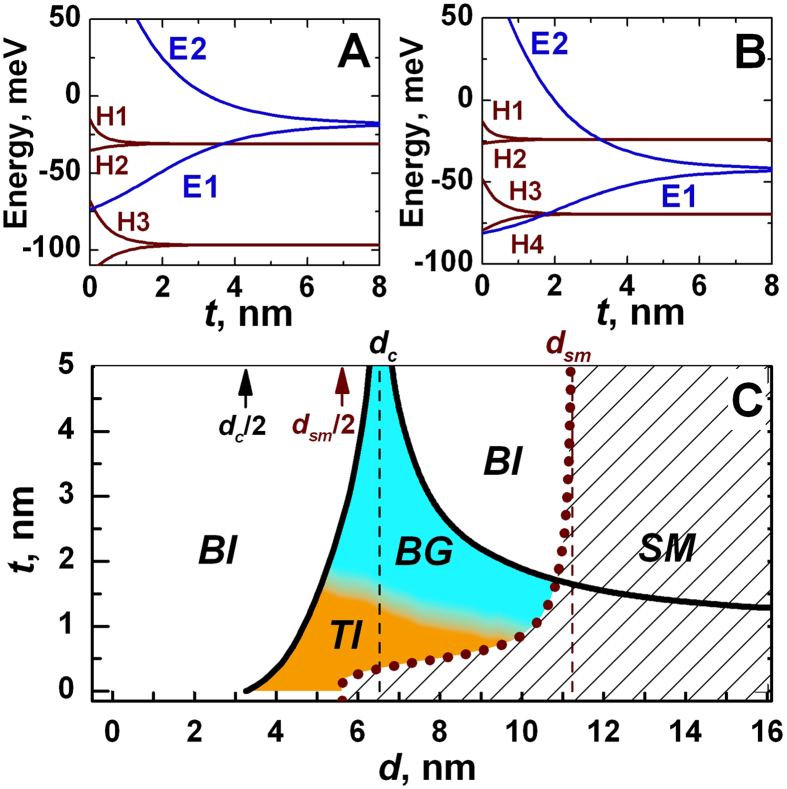
(**A,B**) Energy of *E*1, *E*2 (both in blue) and *H*1, *H*2 (both in red) bands at ***k*** = 0 versus barrier thickness *t* at different quantum well thickness *d*: (**A**) *d*_*c*_/2 < *d* < *d*_*c*_ and (**B**) *d* > *d*_*c*_. (**C**) Phase diagram of double HgTe QW. The values *d*_*c*_ and *d*_*sm*_ correspond to thickness of the single QW, at which Dirac cone and semimetal phase arise respectively. The white-open regions are the band insulator phase, while the white-striped region corresponds to the semimetal phase, when the side maxima of valence subband exceed the bottom of conduction subband. The orange and blue regions conform to topological insulator and BG phase, respectively. The bold black curves correspond to the arising of the Dirac cone at the Γ point. We note that the scales of *d* and *t* in the phase diagram can be efficiently increased by changing *x* and *y* in the alloys of double Hg_*y*_Cd_*1-y*_Te/Cd_*x*_Hg_*1-x*_Te QWs.

**Figure 3:**
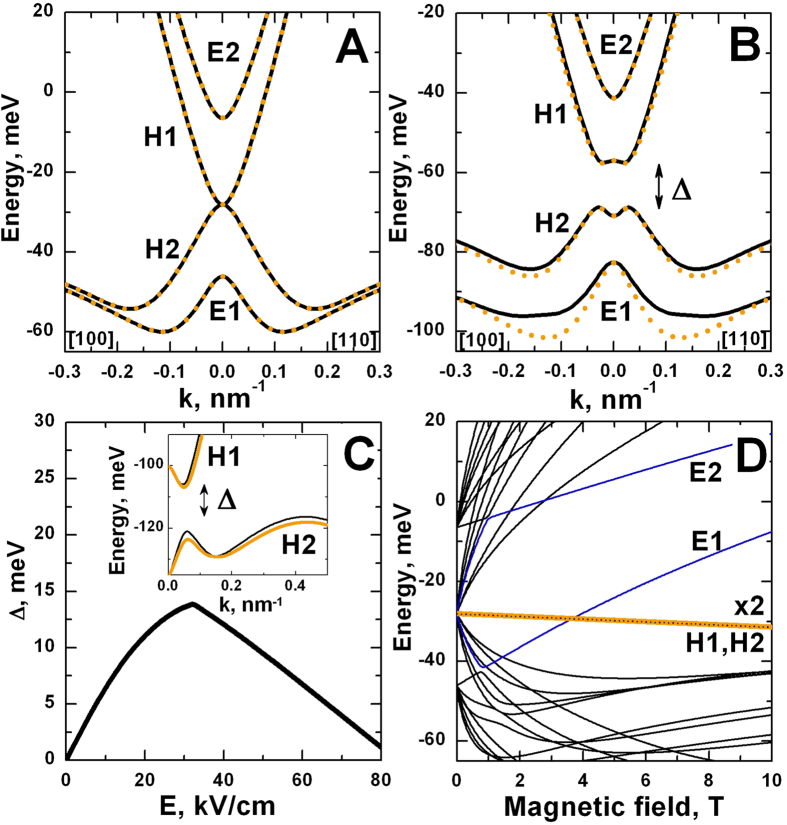
(**A,B**) Energy dispersions for BG phase, implemented at *d* = *d*_*c*_ ≈ 6.5 nm and *t* = 3 nm, in zero electric field (**A**) and in electric field of 20 kV/cm (**B**), oriented perpendicular the QW plane. Solid black and dotted orange curves correspond to different spin states. The presence of electric field not only opens the band gap Δ but also leads to the Rashba spin splitting^22^. (**C**) Bulk band gap as a function of applied electric field. As it is in natural BG^4^,^5^, double HgTe QW in this phase has also electrically-tunable band gap. However, the presence of additional side maximum in the valence subband closes the gap in high electric field. The inset shows energy dispersions in electric field of 50·kV/cm. Solid black and orange curves correspond to different spin states. (**D**) The Landau-level fan chart for BG phase. The zero-mode LL, which has doubled degeneracy order as compared with other levels, is marked by bold orange line. This LL is formed by states of both *H*1 and *H*2 subbands. LLs, containing only the states from *E*1 and *E*2 subbands in high magnetic fields, are given in blue. The crossing between the *E*1 and zero-mode LL, arising at critical magnetic field *B*_*c*_ ~ 3.5 T, leads to the phase transition into normal (non-inverted) band structure, as it is in single HgTe QW^7^.

**Figure 4:**
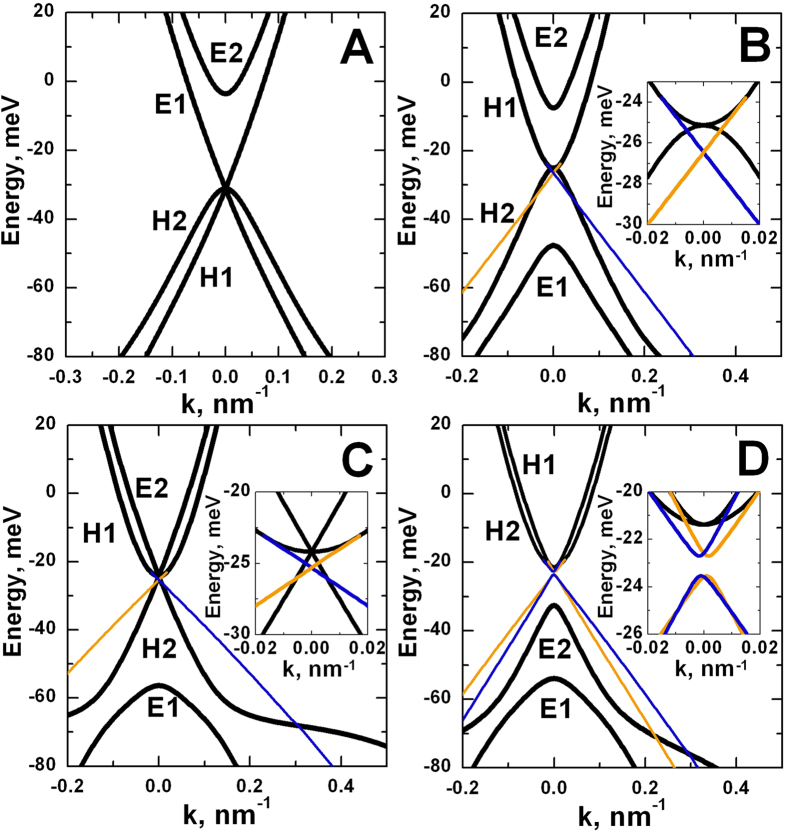
Energy dispersions, calculated by using effective 2D Hamiltonian for (**A**) *M*_*1*_ = 0 and *M*_*2*_ > 0, (**B**) *M*_*1*_ < 0 and *M*_*2*_ > 0 (BG phase), (**C**) *M*_*1*_ < 0 and *M*_*2*_ = 0, (**D**) *M*_*1*_ < 0 and *M*_*2*_ < 0. Other band parameters are given in Supplementary Information. Bulk states are shown in black. Orange and blue curves correspond to the dispersion of the edge states, obtained with open boundary conditions. Kramer’s partners of the edge states, moving in opposite directions, are shown in different colors. The insets show the behavior of the dispersion curves in the vicinity of ***k*** = 0. It is seen from (**A**) and (**C**) that *M*_*1*_ = *0* or *M*_*2*_ = 0 corresponds to topological phase transition with arising of Dirac cone at the Γ point of the Brillouin zone, while each negative values of *M*_*1*_ and *M*_*2*_ results in appearance of additional pair of the helical edge states. The BG phase (**B**) with *M*_*1*_ < 0 and *M*_*2*_ > 0 is nontrivial and characterized by coexistence of gapless bulk states with single pair of the helical edge states. The case of *M*_*1*_ < 0 and *M*_*2*_ < 0 shown in (**D**) is characterized by the presence of two pairs of the edge states. Two edge states with the same spin couple to produce a gap in the spectrum, destroying the QSH effect and putting the system into trivial phase even with inverted band structure in double HgTe QW.
