# Supplementary material for: Phase transitions in two tunnel-coupled HgTe quantum wells: Bilayer graphene analogy and beyond
Source: Sci Rep. 2016 Aug 1;6:30755. doi: 10.1038/srep30755 (PMC4967852; doi:10.1038/srep30755)
Supplement: Supplementary Information [file srep30755-s1.doc]

Phase transitions in two tunnel-coupled HgTe quantum wells.
Bilayer graphene analogy and beyond

S. S. Krishtopenko^1,2^, W. Knap^2^, F. Teppe^2^*

^1^Institute for Physics of Microstructures RAS, GSP-105, 603950, Nizhni Novgorod, Russia

^2^Laboratoire Charles Coulomb (L2C), UMR CNRS 5221, Universite Montpellier, 34095 Montpellier, France

Supplementary materials

8-band Kane model

We consider double HgTe/Cd_0.7_Hg_0.3_Te QW grown on (001)-oriented CdTe buffer and assume that the *z* axis coincides with the crystallographic direction (001), while the *x* and *y* axes correspond to directions (100) and (010), respectively. To correctly account for the influence of nonparabolicity, spin-orbit interaction and lattice-mismatch deformation on electronic states in the double QW, we use the eight band Kane model (*17*). In the basis of Bloch amplitudes for the Γ_6_, Γ_8_ and Γ_7_ bands

$u_{1}(\text{r})=|\Gamma_{6},+1/2\rangle=S\uparrow$,

$u_{2}(\text{r})=|\Gamma_{6},-1/2\rangle=S\downarrow$,

$u_{3}(\text{r})=|\Gamma_{8},+3/2\rangle=(1/\sqrt{2})(X+iY)\uparrow$,

$u_{4}(\text{r})=|\Gamma_{8},+1/2\rangle=(1/\sqrt{6})\left[(X+iY)\downarrow-2Z\uparrow\right]$,

$u_{5}(\text{r})=|\Gamma_{8},-1/2\rangle=-(1/\sqrt{6})\left[(X-iY)\uparrow+2Z\downarrow\right]$,

$u_{6}(\mathbf{r})=|\Gamma_{8},-3/2\rangle=-(1/\sqrt{2})(X-iY)\downarrow$,

$u_{7}(\text{r})=|\Gamma_{7},+1/2\rangle=(1/\sqrt{3})\left[(X+iY)\downarrow+Z\uparrow\right]$,

$u_{8}(\mathbf{r})=|\Gamma_{7},-1/2\rangle=(1/\sqrt{3})\left[(X-iY)\uparrow-Z\downarrow\right]$, (s1)

the Kane Hamiltonian for the envelope function takes the form

where

,

,

,

,

,

,

, $k_{\pm}=k_{x}\pm ik_{y}$, $k_{z}=-i\partial_{z}$. (s2)

Here, [*A*,*B*] = *AB* – *BA* is the commutator, {*A*,*B*} = *AB* + *BA* is the anticommutator for the operators *A* and *B*; *P* is the Kane momentum matrix element; *E_c_*(*z*) and *E_v_*(*z*) are the conduction and valence band edges, respectively; Δ(*z*) is the spin orbit energy; *a_c_* and *a_v_* are the hydrostatic and *b* is the uniaxial deformation potentials; *γ_1_*, *γ_2_*, *γ_3_*, *κ* and *F* describe the interaction with the remote bands, not considered in the Hamiltonian. The terms proportional to non-zero components of the strain tensor *ε_xx_*= *ε_yy_* and *ε_zz_* result from lattice-mismatch strain. From the condition of zero external stress along the (001) direction we get the relation between $\varepsilon_{xx}$and $\varepsilon_{zz}$:

$\varepsilon_{xx}=\frac{a_{CdTe}-a_{L}}{a_{L}}$, $\varepsilon_{zz}=-\frac{2C_{12}}{C_{11}}\varepsilon_{xx}$, (s3)

where *C_ij_* are the elastic constants in each layer, *a_L_* and *a_CdTe_* are the lattice constants of the given layer and CdTe buffer, respectively. In the Kane Hamiltonian, we omit effects of bulk inversion asymmetry introduced by the zinc blende structure of bulk HgTe and CdTe (*S1*) and contribution of spin-orbit interaction into strain-dependent part of the Hamiltonian (*S2*).

Assuming translation invariance in the *xy* plane, envelope function *F_i_*(**r**) for *u_i_*(**r**) Bloch amplitude can be represented as

$F_{i}(\mathbf{r})=\exp(ik_{x}x+ik_{y}y)f_{i}(z)$, (s4)

where *k_x_* and *k_y_* are the wave vector components in the QW plane. As a result, Schrödinger equation with the Kane Hamiltonian and external electric field potential *V*(*z*) is reduced to the following system of differential equations:

$\sum_{j=1}^{8} \left(\mathbf{H}_{ij}+V(z)\delta_{ij}\right)f_{j}(\text{z})=\tilde{E}_{n_{z}}(k_{x},k_{y})f_{i}(\text{z})$, (s5)

where *n_z_* is the electronic subband index. To solve this system, the functions *f_i_*(*z*) are expanded in terms of the complete basis set {$\eta_{\mu}$} of plane waves:

$f_{i}(\text{z})=\frac{1}{\sqrt{L_{z}}}\sum_{\mu=-N}^{N} C_{i}^{(\mu)}\exp(ik_{\mu}z)$, (s6)

where *k_μ_* = 2*πμ*/*L_z_* and *L_z_* is the total width of the DQW structure in *z* direction (in this work, *L_z_* = *L_CdHgTe_^(Top)^* + *2d* + *t* + *L_CdHgTe_^(Back)^*, and *L_CdHgTe_^(Back)^* = *L_CdHgTe_^(Top)^* = 30 nm). In our calculations, *N* defines the accuracy of the solution of the eigenvalue problem, *N* = 100 is good to get convergent results with precision higher than 0.5 %.

The expansion in Eq. s6 leads to a matrix representation of the eigenvalue problem, where the eigenvectors with components $C_{i}^{(\mu)}$ and the corresponding eigenvalues are obtained by diagonalization of matrix $\langle\eta_{\mu}|\mathbf{H}_{ij}+V(z)\delta_{ij}|\eta_{\mu'}\rangle$. By using the plane-wave basis, the matrix elements $\langle\eta_{\mu}|K(z)|\eta_{\mu'}\rangle$, $\langle\eta_{\mu}|K(z)\partial_{z}|\eta_{\mu'}\rangle$, $\langle\eta_{\mu}|\partial_{z}K(z)|\eta_{\mu'}\rangle$ and $\langle\eta_{\mu}|\partial_{z}K(z)\partial_{z}|\eta_{\mu'}\rangle$ can be calculated analytically, where *K*(*z*) is an arbitrary polynomial for each of the QW layers. This also allows one to calculate exactly the matrix element for external electric field potential $\langle\eta_{\mu}|V(z)|\eta_{\mu'}\rangle$. The term *V*(*z*) is determined by,

$\frac{d}{dz}\varepsilon(\text{z})\frac{dV(z)}{dz}=0$, (s7)

and the boundary condition

$\left.\frac{dV(z)}{dz}\right|_{z=-\infty}=E\cdot z$, (s8)

where *E* is electric field strength, *ε*(*z*) is the static dielectric constant, and *z* = 0 corresponds to the right CdHgTe/HgTe interface in Fig. 1.

With the basis expansion method, through the eigenvectors **C** in (s6), we can easily classify the levels. For electronic subband *n_z_*, we define the relative contribution to this level from the basis states in the set *I*:

, (s9)

where *d_I_*(*k_x_*,*k_y_*) is so normalized that if we include all the states in the set *I*, then *d_I_*(*k_x_*,*k_y_*) = 1. In the present work, we will calculate *d_e_* for the contribution from the |Γ_6_, ±1/2〉 states, *d_lh_* for the contribution from the |Γ_8_, ±1/2〉 states, *d_so_* for the contribution from the |Γ_7_, ±1/2〉 states and *d_hh_* for the contribution from the |Γ_8_, ±3/2〉 states. For example, to calculate *d_hh_* from (s9), we let *I* contain *i* =3,6. It is clear that *d_e_*+*d_lh_*+*d_so_*+*d_hh_*= 1 at any values of ***k***. We classify electronic subbands in double HgTe QW as electron-like or hole-like levels by comparing the value of *d_e_*+*d_lh_*+*d_so_* with *d_hh_*. The given subband is the hole-like level if *d_hh_* > *d_e_*+*d_lh_*+*d_so_* at ***k*** = 0. Otherwise, the subbands are classified as electron-like, light-hole-like or spin-off-like levels, according to the dominant component in the sum *d_e_*+*d_lh_*+*d_so_*.

To calculate the energy levels in perpendicular magnetic field **B** = (0, 0, *B*) we use a Peierls substitution

,

, (s10)

and introduce ladder operators *b^+^* and *b*:

$b^{+}=\frac{a_{B}}{\sqrt{2}}k_{+}$, $b=\frac{a_{B}}{\sqrt{2}}k_{-}$. (s11)

where *a_B_* is the magnetic length (*a_B_*^2^ = *cħ*/*eB*), *e* > 0 is the elementary charge and ***A*** is the magnetic vector potential in Landau gauge ***A***= (0, *Bx*,0).

Additionally, the Zeeman term *H_Z_* has to be included in the Hamiltonian. According to (*S3*), *H_Z_* has the form:

$H_{Z}=\mu_{B}B\left(\begin{matrix} 1 & 0 & 0 & 0 & 0 & 0 & 0 & 0 \\ 0 & -1 & 0 & 0 & 0 & 0 & 0 & 0 \\ 0 & 0 & -3\kappa& 0 & 0 & 0 & 0 & 0 \\ 0 & 0 & 0 & -\kappa& 0 & 0 & -\sqrt{2}\kappa& 0 \\ 0 & 0 & 0 & 0 & \kappa& 0 & 0 & -\sqrt{2}\kappa\\ 0 & 0 & 0 & 0 & 0 & 3\kappa& 0 & 0 \\ 0 & 0 & 0 & -\sqrt{2}\kappa& 0 & 0 & -2\kappa& 0 \\ 0 & 0 & 0 & 0 & -\sqrt{2}\kappa& 0 & 0 & 2\kappa\end{matrix}\right)$, (s12)

where *μ_B_* is the Bohr magneton.

We use so-called axial approximation to calculate LLs in HgTe DQW. Within this approximation we keep the in-plane rotation symmetry by omitting the warping term, proportional to (*γ_3_* – *γ_2_*), in *R* (see Eq. s2). As a result, the electron wave function is written as

,

,

, (s13)

where *L_y_* is the sample size along the *y* axis, *H_n_* are the Hermitian polynomials with number *n* (*n* is also the Landau level index and the eigenvalue of the operator *b^+^b*), $\tilde{k}$ is the wave vector projection onto the *y* axis.

For *n* = 0, there is one-component wave function of so-called zero-mode LLs, which are not mixed with other LLs at *n* > 0 and are formed by heavy-hole states |Γ_8_, –3/2〉 only:

. (s14)

The functions *c_6_*(*z*,0,*n_z_*) are satisfied to the following equations:

,

. (s15)

It is clear that the first four terms in $\tilde{H}_{n=0}$ also define energy of hole-like subbands (H1, H2, etc.) at ***k*** = 0 (cf. Eq. s2). Therefore, if the middle barrier thickness *t* is large enough for merging of hole-like subband pairs at ***k*** = 0 (see Fig. 2A, B) and *V*(*z*) = 0, the zero-mode LLs become double-degenerated as compared with other LLs. Thus, the unconventional quantum Hall effect measured in natural BG, should be observed in *all phases* of double HgTe QWs with *t* values, at which H1 and H2 subbands coincide. Non-zero external electric field *V*(*z*) breaks coincidence between H1 and H2 subbands at ***k*** = 0 and, therefore, removes the degeneracy of zero-mode LLs, giving equidistant plateaus in the Hall conductivity.

To solve the Schrödinger equation in magnetic field, we also expand functions *c_i_*(*z*,*n*,*n_z_*), *i* = 1,…8, by a series of plane waves, as it is done in the absence of magnetic field. In Table S1 we list the band parameters used in our calculation. The material parameters are taken from (*17*) except *a_c_*, *a_v_*, *b*, *d* and the estimates for the elastic modules *C_ij_* which are taken from (*S4*). For the band gap *E_g_* = *E_c_* – *E_v_* in Cd_x_Hg_1-x_Te alloy, we used parabolic dependence on *x* between the values in CdTe and HgTe (*S5*), while static dielectric constant is assumed to be *ε* = 20.5 – 15.6·*x* + 5.7·*x*^2^ (*S6*). Other band parameters are considered as piecewise functions along the growth direction and to vary linearly with *x* in the alloy.

**Table S1.** Band parameters for HgTe and CdTe used in calculations.

| Parameters | CdTe | HgTe | Parameters | CdTe | HgTe |
| --- | --- | --- | --- | --- | --- |
| *E_g_*, eV | 1.606 | -0.303 | *E_P_*, eV | 18.8 | 18.8 |
| *E_v_*, eV | -0.57 | 0 | *a*, Å | 6.48 | 6.46 |
| Δ, eV | 0.91 | 1.08 | *a_c_*, eV | -2.925 | -2.380 |
| *F* | 0 | -0.09 | *a_v_*, eV | 0 | 1.31 |
| *γ*_1_ | 1.47 | 4.1 | *b*, eV | -1.2 | -1.5 |
| *γ*_2_ | -0.28 | 0.5 | *C*_11_, 10^11^ din/cm^2^ | 5.62 | 5.92 |
| *γ*_3_ | 0.03 | 1.3 | *C*_12_, 10^11^ din/cm^2^ | 3.94 | 4.14 |
| κ | -1.31 | -0.4 | - | - | - |

Effective 2D Hamiltonian for E1, E2, H1 and H2 levels

We now derive an 8×8 effective 2D Hamiltonian used for qualitative description of quantum phase transitions and corresponding picture of the edge states in double HgTe QWs. Such an approach is valid as long as 2*M_1_* and 2*M_2_* are smaller than the energy separation from considered *E*1, *E*2, *H*1, *H*2 subbands to other excited subbands. Our starting point is the eight-band Kane Hamiltonian in the absence of small terms resulted from bulk inversion asymmetry and strain-dependent part of spin-orbit interaction. Here, we focus on the case, in which structure inversion symmetry holds. Thus, the corresponding effective 2D model describes the double QWs with HgTe layers of equal thicknesses in the absence of electric field. For simplicity, we also keep the in-plane rotation symmetry by adopting the axial approximation mentioned above.

Following the procedure described in (*S7*), we split the Hamiltonian in Eq. (s2) into two parts **H** = **H**_0_(*k_z_*) + **H**_1_(*k_z_*, *k_x_*, *k_y_*), where **H**_0_(*k_z_*) is the Kane Hamiltonian at *k_x_*,*_y_*=0. First, we numerically diagonalize the Hamiltonian **H**_0_(*k_z_*), to obtain the energies and envelope functions *f_i_*(*z*) in Eq. (s2), as well as to as to classify of electronic levels as electron-like *En*, hole-heavy-like *Hn*, light-hole-like *LHn* or spin-off-like *SOn* levels (*n* = 1, 2, …). It is clear from the form of **H**_0_(*k_z_*) that the hole-heavy-like levels at *k_x,y_*=0 are decoupled from the *En*, *LHn* and *SOn* subbands. Therefore, the eigenfunctions of **H**_0_(*k_z_*) are expanded in the basis of Bloch amplitudes as follows:

$|En,+\rangle=f_{1}^{(En,+)}(z)|\Gamma_{6},+1/2\rangle+f_{4}^{(En,+)}(z)|\Gamma_{8},+1/2\rangle+f_{7}^{(En,+)}(z)|\Gamma_{7},+1/2\rangle$,

$|En,-\rangle=f_{2}^{(En,-)}(z)|\Gamma_{6},-1/2\rangle+f_{5}^{(En,-)}(z)|\Gamma_{8},-1/2\rangle+f_{8}^{(En,-)}(z)|\Gamma_{7},-1/2\rangle$,

$|Hn,+\rangle=f_{3}^{(Hn,+)}(z)|\Gamma_{8},+3/2\rangle$,

$$|Hn,-\rangle=f_{6}^{(Hn,-)}(z)|\Gamma_{8},-3/2\rangle$$

$|LHn,+\rangle=f_{1}^{(LHn,+)}(z)|\Gamma_{6},+1/2\rangle+f_{4}^{(LHn,+)}(z)|\Gamma_{8},+1/2\rangle+f_{7}^{(LHn,+)}(z)|\Gamma_{7},+1/2\rangle$,

$|LHn,-\rangle=f_{2}^{(LHn,-)}(z)|\Gamma_{6},-1/2\rangle+f_{5}^{(LHn,-)}(z)|\Gamma_{8},-1/2\rangle+f_{8}^{(LHn,-)}(z)|\Gamma_{7},-1/2\rangle$,

$|SOn,+\rangle=f_{1}^{(SOn,+)}(z)|\Gamma_{6},+1/2\rangle+f_{4}^{(SOn,+)}(z)|\Gamma_{8},+1/2\rangle+f_{7}^{(SOn,+)}(z)|\Gamma_{7},+1/2\rangle$,

$|SOn,-\rangle=f_{2}^{(SOn,-)}(z)|\Gamma_{6},-1/2\rangle+f_{5}^{(SOn,-)}(z)|\Gamma_{8},-1/2\rangle+f_{8}^{(SOn,-)}(z)|\Gamma_{7},-1/2\rangle$. (s16)

Since we hold structure inversion symmetry, the inversion operation **P**, defining the parity of each subband, commutes with the Hamiltonian **H**_0_(*k_z_*). The parity of the subbands is determined by both the envelope functions *f_i_*(*z*) and the Bloch amplitudes in the Γ point. The parities of the envelope functions are obtained through numerical calculations, and are given in Table S2. The parities of the Bloch amplitudes are given by **P**|Γ_6_, ±1/2〉 = – |Γ_6_, ±1/2〉, **P**|Γ_7_, ±1/2〉 = |Γ_7_, ±1/2〉, **P**|Γ_8_, ±1/2〉 = |Γ_8_, ±1/2〉 and, **P**|Γ_8_, ±3/2〉 = |Γ_8_, ±3/2〉. Thus, the parities of the subbands are **P**|*E*1,±〉 = – |*E*1,±〉, **P**|*E*2,±〉 = |*E*2,±〉, **P**|*H*1,±〉 = |*H*1,±〉, **P**|*H*2,±〉 = – |*H*2,±〉, **P**|*LH*1,±〉 = |*LH*1,±〉, **P**|*LH*2,±〉 = – |*LH*2,±〉, and etc.

**Table S2.** Parities of the envelope function components.

| Subband | *E*2,+ | *E*2,– | *E*1,+ | *E*1,– | *LH*1,+ | *LH*1,– |
| --- | --- | --- | --- | --- | --- | --- |
| Even | $f_{4}^{(E2,+)}(z)$, $f_{7}^{(E2,+)}(z)$ | $f_{5}^{(E2,-)}(z)$, $f_{8}^{(E2,-)}(z)$ | $f_{1}^{(E1,+)}(z)$ | $f_{2}^{(E1,-)}(z)$ | $f_{4}^{(LH1,+)}(z)$, $f_{7}^{(LH1,+)}(z)$ | $f_{5}^{(LH1,-)}(z)$, $f_{8}^{(LH1,-)}(z)$ |
| Odd | $f_{1}^{(E2,+)}(z)$ | $f_{2}^{(E2,-)}(z)$ | $f_{4}^{(E1,+)}(z)$, $f_{7}^{(E1,+)}(z)$ | $f_{5}^{(E1,-)}(z)$, $f_{8}^{(E1,-)}(z)$ | $f_{1}^{(LH1,+)}(z)$ | $f_{2}^{(LH1,-)}(z)$ |

| Subband | *H*(2*n*),+ | *H*(2*n*),– | *H*(2*n*-1),+ | *H*(2*n*-1),– |
| --- | --- | --- | --- | --- |
| Even | – | – | $f_{3}^{(H(2n-1),+)}(z)$ | $f_{6}^{(H(2n-1),-)}(z)$ |
| Odd | $f_{3}^{(H(2n),+)}(z)$ | $f_{6}^{(H(2n),-)}(z)$ | – | – |

In accordance with (*S7*), we group the eigenstates of Eq. s16 into two classes. The first class one, marked as class A, includes the basis states of our final effective 2D Hamiltonian {|*E*1,±〉, |*H*1,±〉, |*H*2,±〉, |*E*2,±〉}. In the second class, denoted as class B, we consider the following basis states {|*H*3,±〉, |*H*4,±〉, |*LH*1,±〉, |*LH*2,±〉, |*H*5,±〉, |*H*6,±〉}. All the other subbands of the QW are neglected since they are well separated in energy. The states in both classes are not coupled, since they are eigenstates of Hamiltonian **H**_0_(*k_z_*). However, the presence of **H**_1_(*k_z_*, *k_x_*, *k_y_*) introduces the mixing between the states from classes A and B. To derive an effective 2D Hamiltonian *H_eff_*(*k_x_, k_y_*) for double HgTe, we treat **H**_1_(*k_z_*, *k_x_*, *k_y_*) as a small perturbation and perform a unitary transformation (*S3*) to eliminate the coupling between the states from different classes by applying the second-order perturbation formula

$H_{eff}(k_{x},k_{y})_{m,m'}=E_{m}\delta_{m,m'}+H'_{m,m'}+\frac{1}{2}\sum_{l} H'_{m,l}H'_{l,m'}\left(\frac{1}{E_{m}-E_{l}}+\frac{1}{E_{m'}-E_{l}}\right)$, (s17)

where

,

. (s18)

The summation indices *m*, *m’* correspond to the states in class A, while index *l* is for the states in class B. The Greek indices label envelope function component of the Kane Hamiltonian. We note that accounting for the parity of the envelope functions *f_α_*(*^m^*)(*z*), given in Table S2, greatly simplifies calculation of *H’_m_,_m’_* in the perturbation procedure above.

Ordering the basis states as {|*E*1,+〉, |*H*1,+〉, |*H*2,–〉, |*E*2,–〉, |*E*2,+〉, |*H*2,+〉, |*H*1,–〉, |*E*1,–〉}, after calculating the matrix-elements (s17), we are left with an effective Hamiltonian parameterized in the following way

$H_{eff}(k_{x},k_{y})=\left(\begin{matrix} H(k_{x},k_{y}) & 0 \\ 0 & \Theta H(k_{x},k_{y})\Theta^{-1} \end{matrix}\right)$, (s19)

where Θ is a “time reversal” operator, given by

$\Theta=\left(\begin{matrix} 0 & i\sigma_{y} \\ i\sigma_{y} & 0 \end{matrix}\right)K$, (s20)

with *K* stands for complex conjugation and *σ_y_* is one of the Pauli spin matrices. In (s19) *H*(*k_x_*, *k_y_*) is written as

$H(k_{x},k_{y})=\left(\begin{matrix} \varepsilon_{E1}(k) & -A_{1}k_{+} & R_{1}k_{-}^{2} & S_{0}k_{-} \\ -A_{1}k_{-} & \varepsilon_{H1}(k) & 0 & R_{2}k_{-}^{2} \\ R_{1}k_{+}^{2} & 0 & \varepsilon_{H2}(k) & A_{2}k_{+} \\ S_{0}k_{+} & R_{2}k_{+}^{2} & A_{2}k_{-} & \varepsilon_{E2}(k) \end{matrix}\right)$, (s21)

$k_{\pm}=k_{x}+ik_{y}$,

$\varepsilon_{E1}(k)=C+\frac{\Delta_{H1H2}}{2}+2M_{1}+B_{E1}(k_{x}^{2}+k_{y}^{2})$,

$\varepsilon_{H1}(k)=C+\frac{\Delta_{H1H2}}{2}+B_{H1}(k_{x}^{2}+k_{y}^{2})$,

$\varepsilon_{H2}(k)=C-\frac{\Delta_{H1H2}}{2}+B_{H2}(k_{x}^{2}+k_{y}^{2})$,

$\varepsilon_{E2}(k)=C-\frac{\Delta_{H1H2}}{2}+2M_{2}+B_{E2}(k_{x}^{2}+k_{y}^{2})$.

Band parameters *C*, *M_1_*, *M_2_*, *A_1_*, *A_2_*, *B_E1_*, *B_H1_*, *B_H2_*, *B_E2_*, *Δ_H1H2_*, *R_1_*, *R_2_*, *S_0_* depend on *d*, *t* and material of the QW and barrier layers and are calculated numerically by using envelope function component (integration over *z* axis). Their values are listed in Table S3. We note that if *Δ_H1H2_* = *0* the straightforward calculations results in *B_H1_*= *B_H2_*. Comparison of the bulk electronic subbands, calculated within the eight-band Kane model and Hamiltonian *H_eff_*(*k_x_*, *k_y_*), is given in Fig. S3. One can see good agreement between results from both models at small quasimomentum values.

**Table S3.** Structure parameters involved in effective Hamiltonian *H*(*k_x_*, *k_y_*).

| Panel in Fig. 4 | *d*, nm | *t*, nm | *C*, meV | *Δ_H1H2_*, meV | *M_1_*, meV | *M_2_*, meV | *A_1_*, meV·nm | *A_2_*, meV·nm |
| --- | --- | --- | --- | --- | --- | --- | --- | --- |
| **A** | 6.0 | 6.65 | –31.1 | 0 | 0 | 13.8 | 387 | –373 |
| **B** | 6.5 | 3.0 | –25.1 | 0 | –11.3 | 8.8 | 378 | 358 |
| **C** | 7.5 | 3.28 | –24.2 | 0 | –16.1 | 0 | 358 | –340 |
| **D** | 7.5 | 4.0 | –21.4 | 0 | –16.3 | –2.25 | 360 | –348 |

| Panel in Fig. 4 | *R_1_*, meV·nm^2^ | *R_2_*, meV·nm^2^ | *S_0_*, meV·nm | *B_E1_*, meV·nm^2^ | *B_H1_*, meV·nm^2^ | *B_H2_*, meV·nm^2^ | *B_E2_* meV·nm^2^ |
| --- | --- | --- | --- | --- | --- | --- | --- |
| **A** | 238 | 101 | 0.5 | 1258 | –145 | –145 | 893 |
| **B** | –160 | 152 | –6.5 | 1758 | –115 | –115 | 894 |
| **C** | 478 | 154 | –7.3 | 2302 | –109 | –109 | 1096 |
| **D** | –329 | –107 | 2.3 | 2138 | –152 | –152 | 1253 |

The form (s20) for a “time reversal” operator Θ mimics the one for spin *J* = 3/2. If one re-writes the quasimomentum in the polar coordinate system as *k_x_* = *k·*cos(*θ*), *k_y_* = *k·*sin(*θ*), it is easy to make sure that the following equation holds

$H(k,0)=\exp\left(i\cdot J_{z}\theta\right)\cdot H(k\cos(\theta),k\sin(\theta))\cdot\exp\left(-i\cdot J_{z}\theta\right)$, (s22)

where *J_z_* is one of the spin matrices:

$J_{x}=\frac{1}{2}\left(\begin{matrix} 0 & \sqrt{3} & 0 & 2 \\ \sqrt{3} & 0 & 0 & 0 \\ 0 & 0 & 0 & \sqrt{3} \\ 2 & 0 & \sqrt{3} & 0 \end{matrix}\right)$, $J_{y}=\frac{i}{2}\left(\begin{matrix} 0 & \sqrt{3} & 0 & -2 \\ -\sqrt{3} & 0 & 0 & 0 \\ 0 & 0 & 0 & \sqrt{3} \\ 2 & 0 & -\sqrt{3} & 0 \end{matrix}\right)$, $J_{z}=\frac{1}{2}\left(\begin{matrix} 1 & 0 & 0 & 0 \\ 0 & 3 & 0 & 0 \\ 0 & 0 & -3 & 0 \\ 0 & 0 & 0 & -1 \end{matrix}\right)$. (s23)

Here the matrices are written for the following order of *J_z_* = {1/2, 3/2,-3/2,-1/2}.

Equation (s22) means that the wave function $|S_{k}^{\theta}\rangle$ for the upper block in *H_eff_*(*k·*cos(*θ*),*k·*sin(*θ*)) can be obtained from the initial state along the *x*-axis by the rotation operation $|S_{k}^{\theta}\rangle=\exp\left(-i\cdot J_{z}\theta\right)|S_{k}^{0}\rangle$. The latter clearly resembles that of a four-component spinor describing the spin *J* = 3/2, but arising from the symmetry of E1, H1, H2, E2 subbands. We note that the above rotation operation also implies that the orientation of the pseudo spin ***J*** = (*J_x_*, *J_y_*, *J_z_*) is tied to the quasimomentum ***k_||_*** = (*k_x_*, *k_y_*, *0*). So far our analysis is focused on the upper block of *H_eff_*(*k_x_*, *k_y_*). Since Θ commutes with exp(–*i*·*J_z_θ*), the rotation operation can be applied for spinor of Θ*H_eff_*(*k_x_*, *k_y_*)Θ^-1^ as well.

Using (s23) and the anticommutator {*J_α_*, *J_β_*} = *J_α_J_β_* + *J_β_J_α_*, we can alternatively write for *H*(*k_x_*, *k_y_*)

, (s24)

$-\frac{1}{2}\left(\frac{A_{1}-A_{2}}{\sqrt{3}}+S_{0}\right)\left(k_{x}\left\{J_{x},J_{z}^{2}\right\}+k_{y}\left\{J_{y},J_{z}^{2}\right\}\right)$,

.

where $\tilde{\varepsilon}_{E1}(k)$, $\tilde{\varepsilon}_{H1}(k)$, $\tilde{\varepsilon}_{H2}(k)$, $\tilde{\varepsilon}_{E2}(k)$ are obtained from $\varepsilon_{E1}(k)$, $\varepsilon_{H1}(k)$, $\varepsilon_{H2}(k)$, $\varepsilon_{E2}(k)$ by the following substitution:

$B_{E1}=\tilde{B}_{E1}+\frac{7\sqrt{3}}{12}(R_{1}+R_{2})$, $B_{H1}=\tilde{B}_{H1}+\frac{\sqrt{3}}{4}(R_{1}+R_{2})$,

$B_{H2}=\tilde{B}_{H2}+\frac{\sqrt{3}}{4}(R_{1}+R_{2})$, $B_{E2}=\tilde{B}_{E2}+\frac{7\sqrt{3}}{12}(R_{1}+R_{2})$.

Expression for Θ*H_eff_*(*k_x_*, *k_y_*)Θ^-1^ via the spin 3/2 matrices is obtained from (s24) by the substitution ***J → –J***. Therefore, the electron wave function corresponding to the down block of *H_eff_*(*k_x_*, *k_y_*) is also characterized by fermions described by *H*(*k_x_*, *k_y_*) but with opposite helicity.

The Hamiltonian (s24) looks rather complicated; however, it contains the minimal number of quantities for description of BI, TI and BG phases in DQW HgTe. We note that further simplification, performed, for an example, for BG phase by projecting *H*(*k_x_*, *k_y_*) onto the basic functions of *H*1 and *H*2 subbands at ***k_||_*** = 0, results in the model, which actually does not describe behaviour of LLs in the vicinity of Γ point. In particular, the coincidence between zero-mode LLs, marked as *H*1 and *H*2 in Fig. 3D, is missed in this case. The latter can only be described within the Hamiltonian, directly accounting for the coupling between *E*1, *H*1, *H*2, *E*2 subbands.

Numerical calculation of edge state dispersions

To consider the edge states on a single edge, we deal with a system on a half-plane of *y*≤ 0 and replace *k_y_* by –*i∂_y_* in this case. If *M_1_* < 0, the DQW structure supports edge states, which exponentially decay at *y* → –∞. The Hamiltonian *H_eff_*(*k_x_*, *k_y_*) (s19) is block diagonal, and the eigenvalue problem of the upper and lower blocks can be solved separately. To find the energy spectrum the edge states for the upper block, we solve the Schrödinger equation:

$H(k_{x},-i\partial_{y})\Psi_{edge}(x,y)=E_{edge}\Psi_{edge}(x,y)$ (s25)

and put the boundary condition for the wave function to vanish at *y* = 0. Taking into account the translation invariance along the x axis, the wave function of the edge states has the form

$\Psi_{edge}(x,y)\frac{e^{ik_{x}x}}{\sqrt{L_{x}}}\sum_{n=1}^{4} \alpha_{n}e^{\lambda_{n}y}C_{n}^{(edge)}$, (s26)

where *k_x_* is the wave vector along the edge, *L_x_* is the sample size along the *x* axis, *α_n_* are the coefficients, determined by the boundary conditions, *λ_n_* are the complex-valued reciprocal lengths, and $C_{n}^{(edge)}$ are the position-independent normalized four-component columns.

For a given wave vector *k_x_*, relation *λ_n_*(*E_edge_*) and columns $C_{n}^{(edge)}$ are found from the matrix equation

$H(k_{x},-i\lambda)C^{(edge)}=E_{edge}C^{(edge)}$. (s27)

Note that *λ_n_* in general can be complex, corresponding to a mixture of the edge and bulk states. The energy spectrum of the edge states are found from the condition of wave function decay at *y* → -∞ (by implying Re*λ_n_* > 0) and from the boundary condition at *y* → 0:

$\sum_{n=1}^{4} \alpha_{n}C_{n}^{(edge)}=0$. (s28)

The energy spectrum of the edge states for the lower block in *H_eff_*(*k_x_*, *k_y_*) is found in a similar way. Fig. 4 illustrates dispersions of the bulk and edge states in the vicinity of the Γ point, calculated by using *H_eff_*(*k_x_, k_y_*) Hamiltonian.

Effective model of Michetti et al.

The first attempt to investigate properties of DQW HgTe has been performed by Michetti et al. (*14,15*). For the case of well-separated HgTe layers of thickness close to *d_c_*, they proposed to describe each QW with the BHZ model with an additional tunneling Hamiltonian for interlayer coupling.

According to their approach, the functions for electron-like and hole-like states {|*Ef*,+〉, |*Hf*,+〉, |*Eb*,+〉, |*Hb*,+〉, |*Ef*,–〉, |*Hf*,–〉, |*Eb*,–〉, |*Hb*,–〉}, localized in front (*f*) and (*b*) back HgTe layer, are considered as basis states at ***k_||_*** = 0. In this basis, their effective 8×8 Hamiltonian for DQW has the form:

, (s29)

where tilde means the Hermitian conjugation; $H_{0}^{(f+)}$,$H_{0}^{(f-)}$, $H_{0}^{(b+)}$, $H_{0}^{(b-)}$ are 2×2 BHZ Hamiltonians for the pair of |*Ef*,±〉, |*Hf*,±〉 levels and for the ones of |*Eb*,±〉, |*Hb*,±〉 respectively; *h_T_* is a tunneling Hamiltonian, written as:

$h_{T}=\left(\begin{matrix} \Delta_{E} & \alpha k_{+} \\ \alpha k_{-} & \Delta_{H} \end{matrix}\right)$, (s30)

with

$\Delta_{E}=2\langle Ef+|\mathbf{H}|Eb+\rangle$,

$\Delta_{H}=2\langle Hf+|\mathbf{H}|Hb+\rangle$,

$\alpha k_{+}=2\langle Ef+|\mathbf{H}|Hb+\rangle$. (s31)

Here, $\mathbf{H}$ is the eight-band Kane Hamiltonian for DQW.

The essential problem of the model presented above is that the functions |*Ef*,+〉, |*Hf*,+〉, |*Eb*,+〉, |*Hb*,+〉, |*Ef*,–〉, |*Hf*,–〉, |*Eb*,–〉, |*Hb*,–〉 localized in the (*f*) and (*b*) QWs, do not form the orthogonal basis in the case of coupling between layers. It is clear that the inversion operation **P** should commute with the Hamiltonian for symmetric DQW. Therefore, the wave functions corresponding to the two consecutive levels should have different parities and should be the eigenfunctions of the operation **P**. The latter holds even if these two levels have the same energy.

The states |*Ef*,±〉 and |*Eb*,±〉, as well as |*Hf*,±〉 and |*Hb*,±〉 have the same parity, otherwise (s30) does not occur. Let us demonstrate it. It is clear that

$|Ef,+\rangle=f_{1}^{(Ef,+)}(z)|\Gamma_{6},+1/2\rangle+f_{4}^{(Ef,+)}(z)|\Gamma_{8},+1/2\rangle+f_{7}^{(Ef,+)}(z)|\Gamma_{7},+1/2\rangle$,

$|Eb,+\rangle=f_{1}^{(Eb,+)}(z)|\Gamma_{6},+1/2\rangle+f_{4}^{(Eb,+)}(z)|\Gamma_{8},+1/2\rangle+f_{7}^{(Eb,+)}(z)|\Gamma_{7},+1/2\rangle$,

$|Hf,+\rangle=f_{3}^{(Hf,+)}(z)|\Gamma_{8},+3/2\rangle$,

$|Hb,+\rangle=f_{3}^{(Hb,+)}(z)|\Gamma_{8},+3/2\rangle$,

where $f_{1}^{(Ef,+)}(z)$, $f_{1}^{(Eb,+)}(z)$, $f_{3}^{(Hf,+)}(z)$, $f_{3}^{(Hb,+)}(z)$ have even parity, while the envelope functions $f_{4}^{(Ef,+)}(z)$, $f_{4}^{(Eb,+)}(z)$, $f_{7}^{(Ef,+)}(z)$, $f_{7}^{(Eb,+)}(z)$ are of odd parity. This is also illustrated by Fig. 5 in (*14*). Straightforward calculation with accounting of (s2) results in the following expressions for the matrix elements:

,

,

. (s32)

We note that because the integrand is an odd function of *z*. Taking into account expressions for *T*, *U*, *V*, Δ and $\bar{S}_{\pm}$ (s2), it is easy to see that

$\langle Ef+|\mathbf{H}|Eb+\rangle=\frac{\Delta_{E}}{2}+\alpha_{E}\left(k_{x}^{2}+k_{y}^{2}\right)$,

$\langle Hf+|\mathbf{H}|Hb+\rangle=\frac{\Delta_{H}}{2}+\alpha_{H}\left(k_{x}^{2}+k_{y}^{2}\right)$,

$\langle Ef+|\mathbf{H}|Hb+\rangle=\frac{\alpha k_{+}}{2}$, (s33)

where Δ*_E_*, Δ*_H_*, *α_E_*, *α_H_*, *α* are independent on quasimomentum. Up to the second order in ***k_||_***, expressions (s31) and (s33) coincide, proving that basic functions |*Ef*,±〉 and |*Eb*,±〉, as well as |*Hf*,±〉,and |*Hb*,±〉 have the same parity. As we have mentioned above, the latter means that the functions |*Ef*,+〉, |*Hf*,+〉, |*Eb*,+〉, |*Hb*,+〉, |*Ef*,–〉, |*Hf*,–〉, |*Eb*,–〉, |*Hb*,–〉 do not generate the orthogonal basis.

To overcome this contradiction, one should redefine the basic functions, formed by symmetrical and antisymmetrical combination of initial states |*Ef*,±〉, |*Hf*,±〉, |*Eb*,±〉 and |*Hb*,±〉:

$|E1,\pm\rangle=\frac{|Ef\pm\rangle-|Eb\pm\rangle}{2}$,

$|E2,\pm\rangle=\frac{|Ef\pm\rangle+|Eb\pm\rangle}{2}$,

$|H1,\pm\rangle=\frac{|Hf\pm\rangle+|Hb\pm\rangle}{2}$,

$|H2,\pm\rangle=\frac{|Hf\pm\rangle-|Hb\pm\rangle}{2}$.

As it can be demonstrated, applying this basis to matrix element calculations, similar to (s32), one obtains the Hamiltonian *H_eff_*(*k_x_*,*k_y_*) given by (s21).

Edge states in two tunnel-coupled layers of 2D TI

DQW HgTe at *d* > *d_c_* and large values of *t* can be considered as two tunnel-coupled layers of 2D TI. In this case, it is educative to rewrite *H_eff_*(*k_x_*,*k_y_*) in the following way:

, (s34)

where the first term describes the states in two separated HgTe layers

$\hat{H}_{0}(k_{x},k_{y})=\left(\begin{matrix} H_{0}(k_{x},k_{y}) & 0 & 0 & 0 \\ 0 & \Theta_{2D}H_{0}(k_{x},k_{y})\Theta_{2D}^{-1} & 0 & 0 \\ 0 & 0 & H_{0}(k_{x},k_{y}) & 0 \\ 0 & 0 & 0 & \Theta_{2D}H_{0}(k_{x},k_{y})\Theta_{2D}^{-1} \end{matrix}\right)$ (s35)

with Θ_2_*_D_*= exp(–*iπσ_y_*/2) and 2×2 BHZ Hamiltonian *H_0_*(*k_x_*,*k_y_*). The block matrices on the diagonal are written for the following order of the levels: {|*E*1,+〉, |*H*1,+〉}, {|*H*2,–〉, |*E*2,–〉}, {|*E*2,+〉, |*H*2,+〉}, {|*H*1,–〉, |*E*1,–〉}.

Two other terms in (s34) are interpreted as spin-conserved $\hat{H}_{T}^{(0)}(k_{x},k_{y})$ and spin-dependent $\hat{H}_{T}^{(SF)}(k_{x},k_{y})$ tunneling. They are written as:

$\hat{H}_{T}^{(0)}(k_{x},k_{y})=\left(\begin{matrix} -\delta H_{0}(k_{x},k_{y}) & 0 & 0 & 0 \\ 0 & \Theta_{2D}\delta H_{0}(k_{x},k_{y})\Theta_{2D}^{-1} & 0 & 0 \\ 0 & 0 & \delta H_{0}(k_{x},k_{y}) & 0 \\ 0 & 0 & 0 & -\Theta_{2D}\delta H_{0}(k_{x},k_{y})\Theta_{2D}^{-1} \end{matrix}\right)$,

$\hat{H}_{T}^{(SF)}(k_{x},k_{y})=\left(\begin{matrix} 0 & h_{T}^{(SF)}(k_{x},k_{y}) & 0 & 0 \\ \left(h_{T}^{(SF)}(k_{x},k_{y})\right)^{+} & 0 & 0 & 0 \\ 0 & 0 & 0 & \left(\Theta_{2D}h_{T}^{(SF)}(k_{x},k_{y})\Theta_{2D}^{-1}\right)^{+} \\ 0 & 0 & \Theta_{2D}h_{T}^{(SF)}(k_{x},k_{y})\Theta_{2D}^{-1} & 0 \end{matrix}\right)$,

where “+” refers to the Hermitian conjugation. The explicit forms for $\hat{H}_{0}(k_{x},k_{y})$, $\hat{H}_{T}^{(0)}(k_{x},k_{y})$ and $\hat{H}_{T}^{(SF)}(k_{x},k_{y})$ are obtained from comparison with (s19-s21). Here, we are interesting in qualitative changes in the energy spectrum of the edge states in a clean system if one accounts $\hat{H}_{T}^{(0)}(k_{x},k_{y})$ and $\hat{H}_{T}^{(SF)}(k_{x},k_{y})$ consistently.

In the absence of tunneling, the edge states in double QW consist of two coinciding cones, each resulting from isolated HgTe QW with negative mass parameter. In the presence of spin-conserved tunneling, the cones are splitted into symmetric and anti-symmetric states, as it is schematically shown in Fig. S4A. We note that $\hat{H}_{T}^{(0)}(k_{x},k_{y})$ just renormalizes the parameters of BHZ Hamiltonians, standing on the diagonal of $\hat{H}_{0}(k_{x},k_{y})$ (s35). The coupling between different spin states from different layers, which is described by $\hat{H}_{T}^{(SF)}(k_{x},k_{y})$, leads to the gap opening (in Fig. S4B). In this case, Kramer’s doublets are formed by combination of different spin states from different HgTe layers, as shown in Fig. S4C.

1. M. H. Weiler, in Defects, (HgCd)Se, (HgCd)Te, R. K. Willardson and A. C. Beer, Eds. (Academic Press, New York, 1981), vol. 16, p. 119.
2. T. B. Bahder, Eight-band **k⋅p** model of strained zinc-blende crystals. *Phys. Rev. B* **41**, 11992 (1990).
3. R. Winkler, in *Spin-Orbit Coupling Effects in Two-Dimensional Electron and Hole Systems*, Springer Tracts in Modern Physics (Springer, Berlin, 2003), vol. 191.
4. K. Takita, K. Onabe, S. Tanaka, Anomalous magnetoresistance and band crossing in uniaxially compressed HgTe. *Phys. Status Solidi (b)* **92**, 297 (1979).
5. P. Laurenti, *et al.*, Temperature dependence of the fundamental absorption edge of mercury cadmium telluride. *J. Appl. Phys.* **67**, 6454 (1990).
6. J. D. Patterson, W. A. Gobba, S.L. Lehoczky, Electron mobility in n-type Hg_1−x_Cd_x_Te and Hg_1−x_Zn_x_Te alloys. *J. Mater. Res.* **7**, 2211 (1992).
7. D. G. Rothe, *et al.*, Fingerprint of different spin–orbit terms for spin transport in HgTe quantum wells. *New J. Phys.* **12**, 065012 (2010).


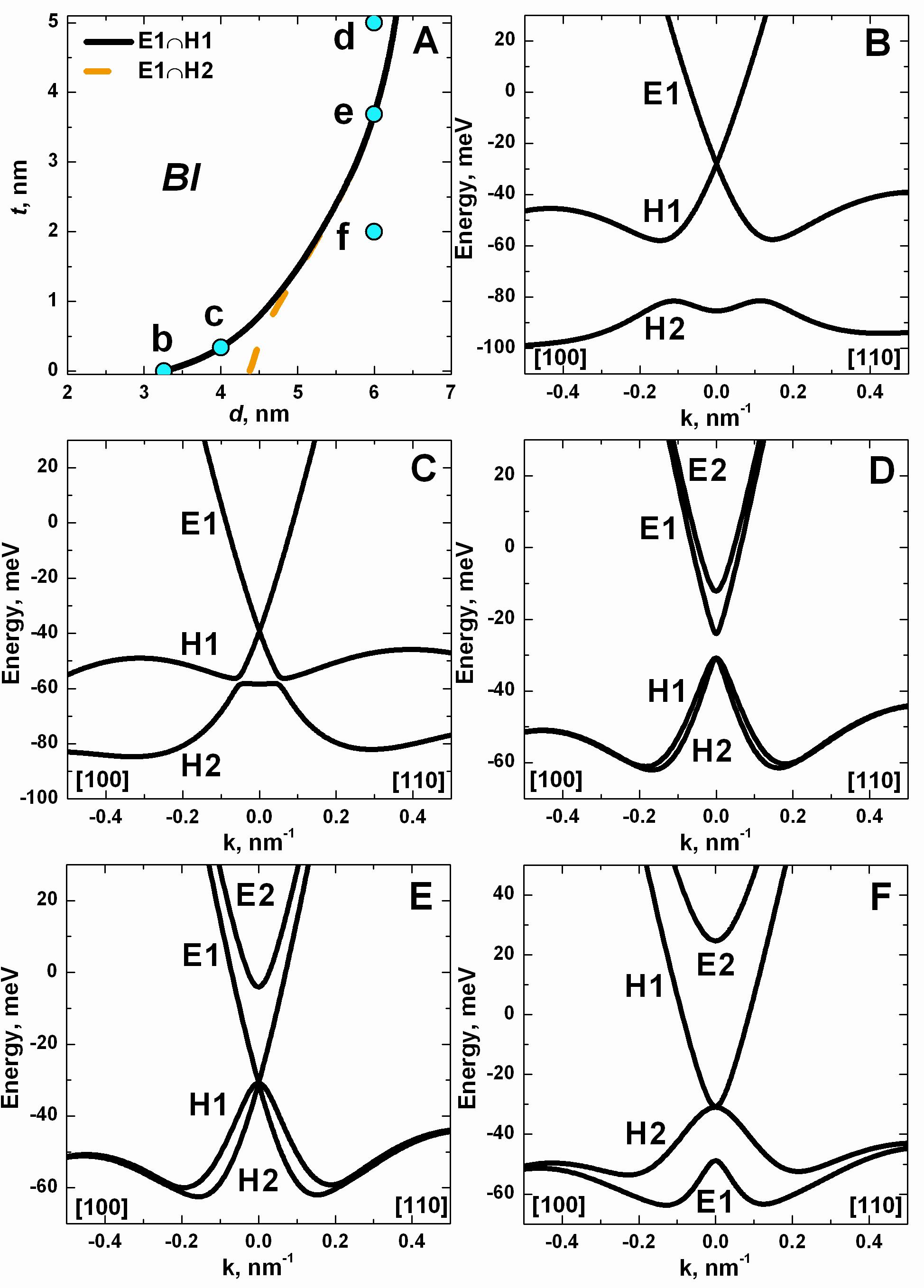


**Fig. S1**. (**A**) A part of the phase diagram showing the QW parameters, at which E1 and H1 subbands are crossed at ***k*** = 0 (bold black curve). The dashed orange curve corresponds to the crossing between E1 and H2 subbands in the Γ point. (**B-F**) Energy dispersions at various *d* and *t*, which values are shown in the panel (**A**) by blue symbols and marked from b to f respectively.


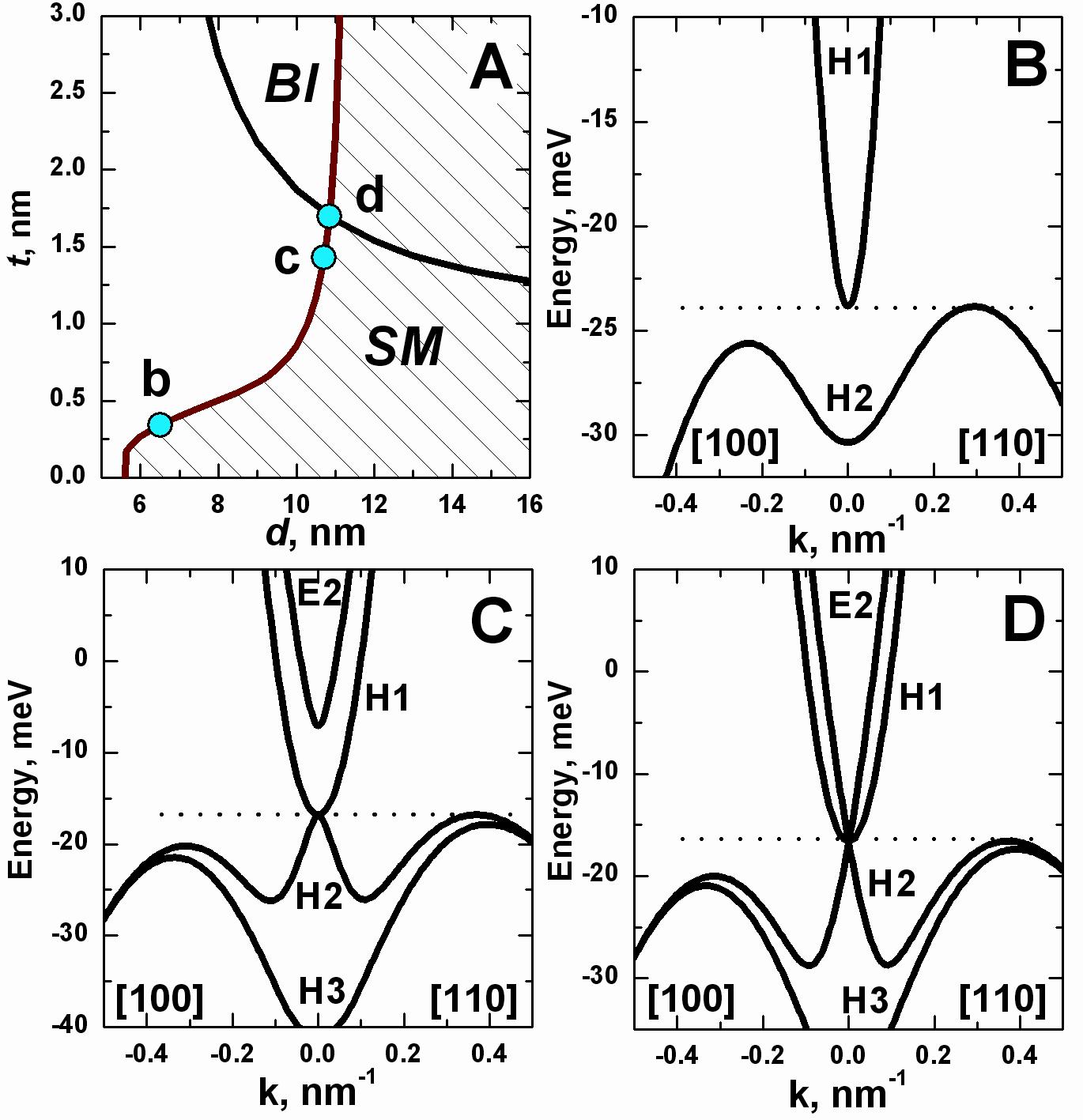


**Fig. S2**. (**A**) A part of the phase diagram, which demonstrates transition into SM phase. The QW parameters, at which a side maximum in the valence band has the same energy as a conduction band bottom, are shown by brown curve. The black curve corresponds to the crossing between *E*2 and *H*2 subbands in the Γ point. (**B-D**) Energy dispersions for the values of *d* and *t*, shown in the panel (**A**) by blue symbols and marked by *b*, *c* and *d* respectively. First electron-like subband *E*1 in all the panels lies significantly below the energy scale.


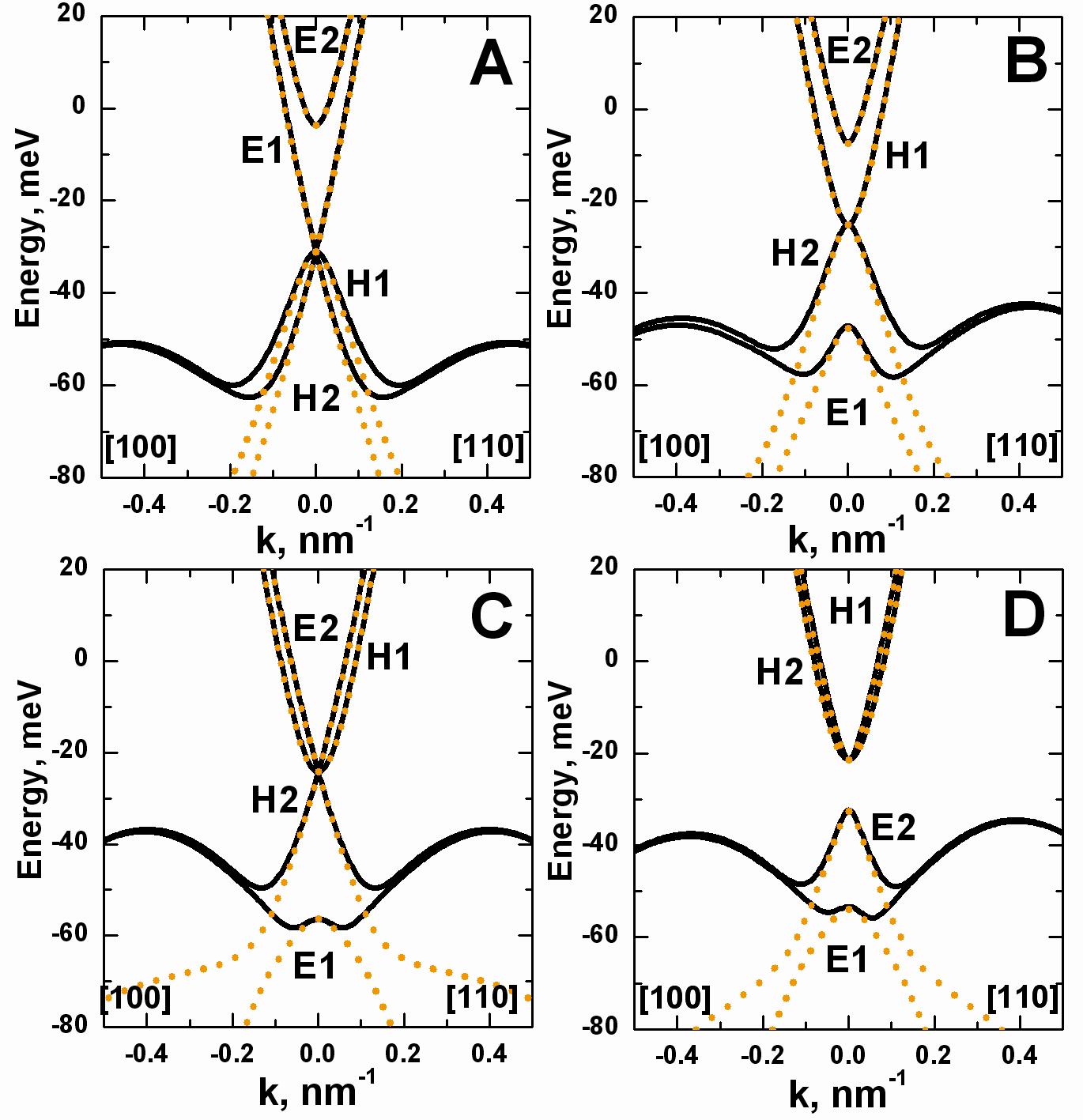


**Fig. S3**. (**A**) Comparison between calculations within the eight-band Kane model (black solid curves) and by using the effective Hamiltonian *H_eff_*(*k_x_*, *k_y_*) (orange dotted curves). The cases (**A**-**D**) are connected with those shown in Fig. 4.


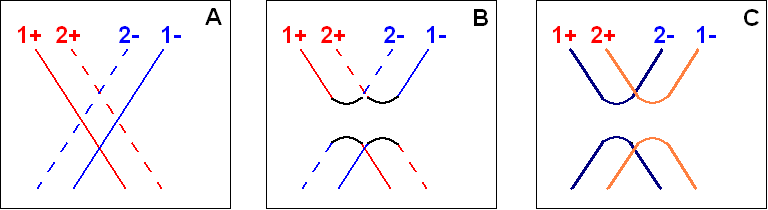


**Fig. S4.** Schematic formation of the edge states in two coupled 2D TI: (**A**) spin-conserved tunneling, (**B**,**C**) spin-dependent tunneling. The solid curves in the panels (A) and (B) present dispersion of the edge states in the first layer, while the dashed curves are the edge states in the second layer. Blue and red colors are for different spin orientation in each layer. The different colored curves in the panel (**C**) correspond to different Kramer’s partners in the whole system.
